# Association of Sexual Attitudes with Sexual Function: General vs. Specific Attitudes

**DOI:** 10.3390/ijerph181910390

**Published:** 2021-10-02

**Authors:** Juan Carlos Sierra, Jennifer Gómez-Carranza, Ana Álvarez-Muelas, Oscar Cervilla

**Affiliations:** Mind, Brain, and Behavior Research Center (CIMCYC), University of Granada, 18011 Granada, Spain; jennifergc@correo.ugr.es (J.G.-C.); alvarezm@ugr.es (A.Á.-M.); ocervilla@ugr.es (O.C.)

**Keywords:** erotophilia, attitude toward sexual fantasies, attitude toward masturbation, sexual function

## Abstract

**Background:** Sexual attitudes are related to the expression of sexuality and have been associated with indicators for sexual health. The main aim of this study was to determine the explanatory capacity of general (i.e., erotophilia) and specific (i.e., toward sexual fantasies and masturbation) sexual attitudes on different sexual functioning dimensions (sexual desire, sexual arousal, lubrication/erection, ability to have an orgasm and orgasm satisfaction). **Methods:** The sample consisted of 2000 heterosexual adults (1044 women, 956 men) aged 18–83 years. **Results:** The explanatory models for women mainly showed that positive attitudes toward sexual fantasies (β range = −0.35, −0.249) and age (β range = −0.111, 0.086) explained sexual function. The models proposed for men revealed a more diverse pattern, although the variable essential for explaining sexual function was a positive attitude toward sexual fantasies (β range = −0.266, −0.097). **Conclusions**: These results indicate that specific sexual attitudes, particularly in relation to sexual fantasies, are more sensitive variables than erotophilia in examining sexual health.

## 1. Introduction

Sexual attitudes are beliefs with a heavy emotional weight that cause subjects to favorably or unfavorably respond to sexual stimuli. Sexual attitudes can be displayed towards sexuality in general (e.g., erotophilia) or certain sexual conducts (e.g., attitude toward sexual fantasies or masturbation). Both types of sexual attitudes significantly determine the way in which people live and express their sexuality, and are associated with different indicators of sexual health, such as skills to prevent sexually transmitted diseases [1], sexual victimization [2] or sexual function [3].

For general attitudes toward sexuality, erotophilia refers to the evaluation of sexual stimuli and is more widely considered and studied as part of the erotophilia–erotophobia continuum. If people come close to the erotophilic extreme, they have positive emotional reactions to sexual stimuli, which are favorably evaluated. Conversely, if people move toward the erotophobic extreme, they feel dissatisfied emotions about sexual stimuli, negatively evaluate them, and so they avoid them [4]. This construct is a traditional research objective in human sexuality research [5]. Erotophilia is related to sexual self-esteem [6], general sexual function [7,8,9,10], sexual desire [11,12,13,14], subjective and objective sexual arousal [15,16], propensity for sexual excitation [17,18], subjective orgasm experience [19], sexual satisfaction [20], sexual fantasies [21], sexual dreams [22], and sexual assertiveness [9,10,23,24], among other constructs. 

One of the most well-studied specific sexual attitudes is the positive attitude toward sexual fantasies. Sexual fantasies are sexual images or thoughts which influence a person’s emotions and/or physiological status. They may occur while practicing sex with a partner or during auto-erotic practices. Most people experience them, and they are generally pleasurable and enriching for sexuality [25]. A positive attitude toward sexual fantasies is related to good sexual function [8,26], sexual satisfaction [27], sexual fantasies [28,29], frequency of sexual fantasies and sexual desire [14,30], positive sexual thoughts [31], sexual assertiveness [23,26], a positive attitude toward masturbation [32], intensity of the subjective orgasm experience in the masturbation context [33] and sexual guilt [34,35]. 

Another attitude toward certain sexual conduct, and one which is interesting for studying sexual health, is the attitude toward masturbation. Masturbation is traditionally considered an unacceptable conduct in some domains (e.g., the Jewish–Christian religion). Consequently, we still note the impact of this tradition as it is still stigmatized today [36,37]. Indeed, a negative attitude toward masturbation is associated with sexual guilt [34,35,38], less frequent orgasms while practicing masturbation [39], less subjective sexual arousal [40], less frequent masturbation [32,41], less solitary sexual desire and a worse sexual function [32].

In the clinical context (e.g., sex therapy), it is usual to assess and listen to the sexual attitudes of patients with sexual dysfunctions. However, it is unclear whether it is more relevant to address general sexual attitudes (e.g., erotophilia) or specific sexual attitudes (e.g., toward sexual fantasies or masturbation). Very few studies have jointly dealt with the association between a general attitude toward sexuality with certain specific attitudes toward given sexual conducts and other sexuality dimensions. One exception is the study by Santos-Iglesias et al. [23], which examined the role of sexual desire, arousal, sexual attitudes and the abuse suffered in a relationship to explain sexual assertiveness. Their results indicated that having an attitude toward a specific sexual conduct (sexual fantasies in their case) was more powerful than erotophilia for predicting sexual assertiveness. Sierra et al. [26] recently reported that a positive attitude toward sexual fantasies, unlike erotophilia, is capable of explaining sexual function in both men and women. More recently, Sierra et al. [2] reported that attitudes toward sexual fantasies, unlike erotophilia, distinguished women who had suffered physical and non-physical abuse by a partner from those who had not endured such experiences. 

In short, general and specific sexual attitudes have normally been covered separately in the scientific literature when studying their relation to other sexuality dimensions, and no studies have examined how both types of attitudes act in relation to constructs like the different sexual function components. This is a relevant matter in clinical sexology because patients’ sexual attitudes tend to be contemplated in sexual therapy to make them positive for the patients [42,43]. Therefore, the present study was conducted to extend knowledge about sexual attitudes in the sexual function context. The main objective was to determine the association that erotophilia, positive attitudes toward sexual fantasies and negative attitudes toward masturbation had with different sexual function components: desire, arousal, erection/vaginal lubrication, ability to have an orgasm and orgasm satisfaction. Due to gender differences evidenced in both sexual attitudes [9,26,30] and sexual functions [7], this association was examined in men and women separately. We hypothesized that a general attitude toward sexuality (erotophilia), and specific attitudes toward sexual fantasies and masturbation, would have a different relevance in explaining sexual function, with more importance attached to attitudes toward specific sexual conducts (sexual fantasies and masturbation), as studies into other constructs have demonstrated [2,23,26].

## 2. Materials and Methods

### 2.1. Participants

The sample consisted of 2000 heterosexual Spanish adults (1044 women, 956 men) aged between 18 and 83 years (*M* = 40.12; *SD* = 12.48). We determined this sample size based on a 97% confidence level and a 3% error estimation. Incidental sampling by quotas according to age was performed: 18–34 (*n* = 699), 35–49 (*n* = 670) and 50 years and older (*n* = 631). The inclusion criteria were: (a) aged 18 years or older; (b) Spanish nationality; (c) heterosexual orientation; (d) current sexual activity with another person of the opposite sex. Table 1 presents the sample’s socio-demographic characteristics. 

### 2.2. Instruments

The socio-demographic and sexual history questionnaire collected data about sex, age, nationality, sexual orientation, level of education, sexual activity, number of sexual partners, partner relationships and religiosity. 

The Spanish version of Sexual Opinion Survey-6 (SOS-6) [9] evaluated erotophilia with six items (e.g., I personally find that thinking about engaging in sexual intercourse is arousing) answered on a 7-Likert scale from 1 (*totally disagree*) to 7 (*totally agree*). Higher scores indicated more erotophilia. These scores indicated adequate evidence for internal consistency reliably (α = 0.74) and suitable evidence for validity based on the relation to other variables (sexual satisfaction, sexual desire, sexual function, sexual assertiveness and positive attitude toward sexual fantasies; [9,15]). In this study sample, the ordinal alpha values were 0.81 in women and 0.85 in men. 

The Spanish version of Hurlbert Index of Sexual Fantasy (HISF) [26] had 10 items (e.g., I think sexual fantasies are healthy) which evaluated the extent of positive attitudes toward sexual fantasies on a Likert scale ranging from 0 (*never*) to 4 (*all the time*). Higher scores indicated a more positive attitude toward sexual fantasies. Its internal consistency reliably was 0.94 and it presented adequate evidence for validity with other similar measures. In this study, the ordinal alpha values were 0.88 in women and 0.87 in men. 

The Spanish version of Negative Attitudes Toward Masturbation Inventory (NATMI) [32]. It was made up of 10 items (e.g., Masturbation in an adult is juvenile and immature) answered on a 5-point Likert scale from 1 (*not at all true from me*) to 5 (*extremely true for me*). Higher scores indicated a more negative attitude toward masturbation. Its internal consistency reliably was high (ordinal alpha of 0.95), and it presented adequate evidence for discriminant and convergent validity. In this sample, the ordinal alpha value was 0.95 for both women and men. 

The Spanish version of the Arizona Sexual Experience Scale (ASEX) [44] was validated in the Spanish population by Sánchez-Fuentes et al. [7]. It comprised of six items answered on a 6-point Likert scale ranging from 1 (*good functioning*) to 6 (*bad functioning*). It assessed sexual function in the last 7 days during sexual intercourse, in terms of desire, arousal, erection (in men)/vaginal lubrication (in women), ability of having an orgasm and orgasm satisfaction. Higher scores indicated a worse sexual function. It presents suitable psychometric properties, with a Cronbach´s alpha value of 0.79 in women and 0.80 in men, and adequate evidence for this validity. In this study sample, the ordinal alpha values were 0.83 in women and 0.81 in men. 

### 2.3. Procedure

Data collection was completed with an online survey on Facebook^®^ (Facebook, Inc, California, United States) from January to March 2020 using the LimeSurvey^®^ software (SourceForge.net, Hamburgm, Germany). This method was normally used in research works about sexual behaviors. There were no differences between the data obtained by this system and those acquired by the traditional paper and pencil methods [45,46]. The IP address was controlled, as were automatic responses to avoid automated responses. The participants accessed the survey by answering a security question consisting of a random arithmetic question. All the responses were examined in detail to control for anomalous patterns. Anonymity and confidentiality were guaranteed. Participation was voluntary and the participants filled in an informed consent form that described the type of study, and provided information on data privacy and confidentiality. The study was previously approved by the Human Research Ethics Committee of the University of Granada.

### 2.4. Data Analysis

The analyses were performed using the R^®^ environment (version 3.6.3) (The R Fundation, Vienna, Austria) [47] with its RStudio^®^ interface (version 1.2.5042) (RStudio PBC, Vienna, Austria) [48]. First, to process any missing data, an algorithm for non-parametric distributions was applied to create a random forest model for each variable by means of the other variables in the database. The missing values were input using the missForest package (versión 1.4) [49]. A MANCOVA was performed to examine the effect of sex on sexual attitudes (erotophilia, positive attitude toward sexual fantasies and negative attitude toward masturbation) and sexual function dimensions (desire, arousal, ability to have an orgasm and orgasm satisfaction), with age, level of education, having a partner, frequency of attending religious events and frequency of praying in private as covariables. Given the found differences per sex, the following analyses were carried out for the sample of women and men separately. The explanatory capacity of age (given its relation to sexual function), as well as sexual attitudes on each sexual function component, were examined by multiple linear regression using the enter method. 

## 3. Results

### 3.1. Sex-Differences in Sexual Attitudes and Sexual Function

Age (Wilk’s lambda = 0.98; F(7, 1827) = 5.46, *p* < 0.001; ƞ2 = 0.02), level of education (Wilk’s lambda = 0.98; F(7, 1827) = 5.36, *p* < 0.001; ƞ2 = 0.02), having a partner (Wilk’s lambda = 0.99; F(7, 1827) = 2.91, *p* < 0.01; ƞ2 = 0.011), frequency of attending religious events (Wilk’s lambda = 0.97; F(7, 1827) = 9.19, *p* < 0.001; ƞ2 = 0.034) and frequency of praying in private (Wilk’s lambda = 0.98; F(7, 1827) = 4.46, *p* < 0.001; ƞ2 = 0.017) were significant multivariate covariates. Sex had a major effect on sexual attitudes and sexual function (Wilk’s lambda = 0.88; F(7, 1827) = 35.0, *p* < 0.001; ƞ2 = 0.118). The intersubject effect on sexual attitudes and sexual function was shown in Table 2. The significant differences per sex were found in specific sexual attitudes, but not in erotophilia, and also appeared in the sexual function dimensions, except in orgasm satisfaction. The effect sizes were medium, except in negative attitude toward masturbation, where they were small. Women showed a more positive attitude toward sexual fantasies than men, whereas men reported a more negative attitude toward masturbation than women. Mens’ scores for the sexual function dimensions indicated a better sexual function in general.

### 3.2. Regression Models

For women, significant models were obtained for desire (F (4, 1039) = 45.84; *p* < 0.001), arousal (F (4, 1039) = 39.98; *p* < 0.001), vaginal lubrication (F (4, 1039) = 24.62; *p* < 0.001), ability to have an orgasm (F (4, 1039) = 2.79; *p* < 0.001) and orgasm satisfaction (F (4, 1039) = 25.14; *p* < 0.001). In the first model, age (β = 0.086) and positive attitudes toward sexual fantasies (β = −0.350) explained 15% of the variance for sexual desire. The variables in the second model were age (β = 0.077) and positive attitudes toward sexual fantasies (β = −0.340), which accounted for 13% of the variance for sexual arousal. In the third model, positive attitudes toward sexual fantasies (β = −0.269) was the only variable, and it accounted for 9% of the variance for vaginal lubrication. In the fourth model, once again age (β = −0.111) and positive attitudes toward sexual fantasies (β = −0.249) explained 8% of the variance for the ability to have an orgasm. Finally, in the last model, positive attitudes toward sexual fantasies (β = −0.284) and negative attitudes toward masturbation (β = 0.067) accounted for 9% of the variance for orgasm satisfaction.

In men, significant models were also obtained for desire (F (4, 951) = 24.75; *p* < 0.001), arousal (F (4, 951) = 26.49; *p* < 0.001), erection (F (4, 951) = 36.15; *p* < 0.001), ability to have an orgasm (F (4, 951) = 18.24; *p* < 0.001) and orgasm satisfaction (F (4, 951) = 20.47; *p* < 0.001). In the first two models, only positive attitudes toward sexual fantasies explained 9% of the variance for sexual desire (β = −0.245) and 10% for sexual arousal (β = −0.266). In the third model, the variables that explained 13% of the variance for erection were age (β = 0.298), erotophilia (β = −0.081), positive attitudes toward sexual fantasies (β = −0.097) and negative attitudes toward masturbation (β = 0.093). In the fourth model, the variables that accounted for 7% of the variance for the ability to have an orgasm were erotophilia (β = −0.133) and positive attitudes toward sexual fantasies (β = −0.144). In the last model, both positive attitudes toward sexual fantasies (β = −0.184) and negative attitudes toward masturbation (β = −0.117) explained 8% of the variance for orgasm satisfaction. The variance inflation factors (VIF) values showed no multicollinearity problems in any of the models for the sexual function dimensions of both men and women (see Table 3 and Table 4).

## 4. Discussion

As far as we are aware, the scientific literature contains no studies that jointly examine the role of general and specific sexual attitudes to explain sexual function. Several research works focused instead on the relation between some attitudes and certain sexual response dimensions, such as desire [12] or orgasm [19], and very few have jointly examined the association of the two types of attitudes (general vs. specific) with other sexual health-related conducts [2,23,26]. For this reason, the objective of the present study was to analyze the capacity of both types of attitudes to explain each sexual function component in women and men. To do so, the general and specific sexual attitudes, with better evidence of their relation to sexual health, were considered: erotophilia, such as a general and positive attitude toward sexual fantasies, and a negative attitude toward masturbation as a specific attitude.

First, the differences between men and women in the study object variables were examined. Congruent differences were generally found compared to previous research. Women reported a worse sexual function than men for all the sexual response components, which agrees with other studies that applied similar measures to populations such as our study population [7,8,50], with the exception of orgasm satisfaction. Similar to other studies, women took a less positive attitude toward sexual fantasies [8,26,28] and a less negative attitude toward masturbation [36,51] than men. Several studies stress the importance of differences per sex in analyzing attitudes and sexual conducts, and often show that men are more prone to contemplate positive aspects of sexuality, such as taking a more positive attitude toward sexual fantasies [30] or better sexual function [8,50]. Women tended to employ masturbation as a strategy to relax or cope with stress, as well as a source of pleasure, rather than a conduct to compensate for not having a partner or as a substitution for sexual intercourse, which was the case for men [37]. This finding might explain why women take a less negative attitude toward masturbation than men. 

It is worth pointing out the lack of significant differences between men and women regarding erotophilia, which tends to be traditionally expressed in the Spanish population [9,23,52,53]. The fact that both men and women take a similar erotophilic attitude could reflect a tendency toward egalitarian views for both sexes regarding sexuality, but could also be the result of using an invariate measure per sex like that herein employed and, unlike other previous works to assess erotophilia, which could eliminate this measure’s possible biases [15]. Finding no differences per sex in orgasm satisfaction coincides with other studies, which found no differences in sexual satisfaction between men and women [27,54]. The fact that significant differences appeared in the ability to have an orgasm, but not in orgasm satisfaction, seems to indicate that orgasmic capacity and sexual satisfaction do not always go hand in hand in women, as previously evidenced in the elderly [8].

For the association of both types (general and specific) of sexual attitudes with different sexual function components (desire, arousal, erection/vaginal lubrication, ability to have an orgasm, orgasm satisfaction), we found that sexual function for both men and women was basically explained by taking a positive attitude toward sexual fantasies and was significant for all its dimensions. In other words, taking a positive sexual attitude toward sexual fantasies was associated with more sexual desire and arousal, better vaginal lubrication/erection capacity, the ability to have an orgasm, and more orgasm satisfaction. These results fall in line with those reported by Sierra et al. [26]. The orgasm satisfaction explanation is also related to a negative attitude toward masturbation, but less significantly than attitude toward sexual fantasies insofar as negative attitude toward masturbation is associated with less orgasm satisfaction. The recent study by Cervilla et al. [32] indicated that those people with a negative attitude toward masturbation reported less orgasm satisfaction in sexual relations than those with a more positive attitude. These results indicate a differential effect of both specific sexual attitudes about sexual response components. The preferred attitudes toward fantasies favored all the sexual function dimensions of both men and women, but the attitudes toward masturbation only did so regarding orgasm satisfaction for both sexes, and for erections for men. The fact that men attached more importance to attitudes toward masturbation than women could be associated with men taking a more negative attitude toward this conduct, compared to women [32]. These results support the relevance of sexual fantasies as a central sexual function component [25]. The DSM-5 [55] considers that having few or no erotic thoughts or fantasies is one of the diagnostic criteria for not only feminine sexual interest/excitation disorder, but also for hypoactive masculine sexual desire. 

As for taking a general attitude toward erotophilia, we observed that while women had no explanatory capacity for sexual function components, men played a secondary role in accounting for erection capacity and the ability to have an orgasm; that is, men with an erotophilic attitude would be associated with a better erection and orgasm capacity. These results supported the proposed hypothesis and, based on previous studies [2,23,26] of erotophilia and attitudes toward sexual fantasies and masturbation, took different weights to explain sexual function, with more importance attached to specific attitudes. These findings evidenced that specific sexual attitudes (particularly positive attitudes toward sexual fantasies) could be more sensitive variables compared to having a general attitude toward sexuality (i.e., erotophilia) to examine sexual health.

Age is seen as a predictor variable because its relation to sexual function is well-known [50,56]. In light of the results herein obtained, the association between age and sexual function dimensions were not presented consistently. Age can show an explanatory capacity for the desire and arousal capacities in women, and for the erection capacity in men. Sexual function becomes more difficult with age [50,57,58], which most likely has something to do with its effect on the physiology of sexual response, especially the lack of hormones which affect women’s sexual desire and men’s erection capacity.

It should be noted that the study sample was composed only of people whose sexual relations were exclusively heterosexual. This fact does not reflect the sexual reality and responds uniquely to the methodological reasons related to the instruments used in the evaluation of sexual attitudes and sexual function, which are validated in the Spanish heterosexual population. Future studies should be interested in their validation in other populations and in examining the invariance of their measures, to be able to integrate sexual diversity into the research. We are aware that sexuality is more diverse; it should not be considered exclusively as sexual intercourse between persons of two different sexes.

Despite the sample being large, this study presents a series of limitations that must be taken into account when interpreting its results. First, patients were selected by non-probabilistic sampling using an online survey only, which meant that users had to have a social network. Second, the sample was formed by people mostly with a university education. Therefore, these limitations must be taken into account to generalize the results. On the other hand, the aim of this study was to explore sexual function in the context of heterosexual intercourse, but it should be emphasized that sexuality is broader and should not be simplified as such.

## 5. Conclusions

By way of conclusion, specific sexual attitudes, especially positive attitudes toward sexual fantasies, are more relevant than a general attitude toward sexuality (erotophilia) to explain sexual function. These results could contribute to extend the knowledge regarding the effect of attitudes on sexual function, and in both the clinical domain and research. First, different models were obtained for both men and women to explain all the sexual function dimensions using different types of sexual attitudes. Second, assessing not only sexual function, but also the variables associated with it, is essential in clinical practice. Thus, professionals must evaluate those attitudes that may play a relevant role in sexual problems, because this is a central part of the interventions in both sexual education and changes in sexual attitudes [42,43,59,60,61]. This means that therapies must focus on sexual function-related elements, particularly on sexual attitudes. Those patients with sexual dysfunctions who seek therapy tend to take more negative attitudes toward different aspects of sexuality. Therefore, they must be dealt with by focusing on the most relevant attitudes in light of the results obtained herein. The findings of this study are also interesting for prevention programs. For example, interventions in negative attitudes toward masturbation are included in sexual education programs to improve different sexual health aspects [62,63]. Therefore, sexual education can promote positive sexual attitudes to do away with, for example, gender roles [45,64] or discrimination against sexual minorities [65,66] which limit sexual health. Therefore, these findings can help effective therapies and programs to be developed that promote, in particular, sexual wellbeing and quality of life in general.

## Figures and Tables

**Table 1:** Socio-demographic characteristics and differences between women and men.

	Total *N* = 2000	Women *N* = 1044	Men *N* = 956	*t*/χ^2^	Cohen’s d
Age (M, SD)	40.12 (12.48)	39.04 (12.25)	41.29 (12.63)	4.05 ***	0.18
Level of education (n, %)				7.29 *	
Primary Education	108 (5.60)	56 (5.60)	52 (5.60)		
Secondary Education	670 (34.70)	319 (31.90)	351 (37.70)		
University degree (ongoing or completed)	1152 (59.70)	624 (62.50)	528 (56.70)		
Number of sexual partners (*M*, *SD*)	11.91 (19.64)	11.61 (16.13)	12.24 (22.88)	0.69	
Currently have a partner (*n*, %)				25.25 ***	
Yes	1626 (81.30)	805 (77.10)	821 (85.90)		
No	374 (18.70)	239 (22.90)	135 (14.10)		
Frequency of attending religious events				11.74	
More than once a day	2 (0.1%)	1 (0.1%)	1 (0.1%)		
Once a day	3 (0.2%)	2 (0.2%)	1 (0.1%)		
A few times a week	17 (0.9%)	13 (1.4%)	4 (0.4%)		
Once a week	54 (2.8%)	28 (3%)	26 (2.6%)		
A few times a month	75 (3.9%)	41 (4.4%)	34 (3.4%)		
Once a month	22 (1.1%)	7 (0.8%)	15 (1.5%)		
Less than once a month	445 (23.1%)	225 (24.2%)	220 (22.1%)		
Never	1309 (67.9%)	613 (65.9%)	696 (69.8%)		
Frequency of praying in private				11.7	
More than once a day	54 (2.8%)	29 (3.1%)	25 (2.5%)		
Once a day	97 (5%)	58 (6.3%)	39 (3.9%)		
A few times a week	106 (5.5%)	48 (5.2%)	58 (5.8%)		
Once a week	10 (0.5%)	3 (0.3%)	7 (0.7%)		
A few times a month	100 (5.2%)	40 (4.3%)	60 (6%)		
Once a month	20 (1%)	7 (0.8%)	13 (1.3%)		
Less than once a month	205 (10.6%)	97 (10.5%)	108 (10.8%)		
Never	1340 (69.4%)	646 (69.6%)	694 (69.1%)		

*Note*. * *p* < 0.05; *** *p* < 0.001.

**Table 2:** Comparison of sexual attitudes and sexual function between women and men.

	Women (*n* = 1044)	Men (*n* = 956)			
	M (SD)	M (SD)	F(6, 1833)	*p*	ƞ^2^
Erotophilia	37.75 (4.81)	37.67 (5.19)	0.29	0.0592	-
Positive attitude toward sexual fantasies	29.76 (6.64)	32.45 (6.03)	88.56	<0.001	0.046
Negative attitude toward masturbation	10.78 (2.24)	11.25 (2.99)	12.46	<0.001	0.007
Sexual desire	2.92 (1.15)	2.56 (0.92)	53.81	<0.001	0.029
Sexual arousal	2.97 (1.08)	2.64 (0.88)	52.0	<0.001	0.028
Ability to have an orgasm	2.80 (1.18)	2.39 (0.92)	−61.96	<0.001	0.033
Orgasm satisfaction	2.02 (1.14)	1.97 (0.91)	0.13	0.724	-

*Note*. M = mean; SD = Standard deviation; ƞ^2^ = partial eta square.

**Table 3:** Hierarchical regression for sexual function in women.

Predictors		B	SE	β	95%CI	t	*p*	VIF	$R^{2}$
Model 1. Sexual desire	Age	0.008	0.003	0.086	0.003, 0.013	3.002	0.003	1.009	0.15
	Erotophilia	−0.013	0.008	−0.052	−0.028, 0.003	−1.604	0.109	1.271	
	Positive attitudes toward sexual fantasies	−0.061	0.006	−0.350	−0.072, −0.050	−10.944	<0.001	1.249	
	Negative attitudes toward masturbation	0.001	0.015	0.002	−0.030, 0.031	0.054	0.957	1.092	
Model 2. Sexual arousal	Age	0.007	0.003	0.077	0.002, 0.012	2.657	0.008	1.009	0.13
	Erotophilia	−0.009	0.007	−0.038	−0.023, 0.006	−1.177	0.239	1.271	
	Positive attitudes toward sexual fantasies	−0.056	0.005	−0.340	−0.066, −0.045	−10.536	<0.001	1.249	
	Negative attitudes toward masturbation	−0.007	0.015	−0.014	−0.036, 0.022	−0.453	0.651	1.092	
Model 3. Vaginal lubrication	Age	0.005	0.003	0.054	0.000, 0.010	1.827	0.068	1.009	0.08
	Erotophilia	−0.006	0.008	−0.025	−0.021, 0.009	−0.745	0.456	1.271	
	Positive attitudes toward sexual fantasies	−0.044	0.005	−0.269	−0.055, −0.034	−8.114	<0.001	1.249	
	Negative attitudes toward masturbation	0.013	0.015	0.026	−0.017, −0.043	0.854	0.393	1.092	
Model 4. Ability to have an orgasm	Age	−0.011	0.003	−0.111	−0.016, −0.005	−3.714	<0.001	1.009	
	Erotophilia	0.001	0.008	0.005	−0.015, 0.017	0.135	0.893	1.271	
	Positive attitudes toward sexual fantasies	−0.044	0.006	−0.249	−0.056, −0.033	−7.481	<0.001	1.249	
	Negative attitude toward masturbation	0.026	0.016	0.049	−0.006, 0.058	1.571	0.116	1.092	
Model 5. Orgasm satisfaction	Age	−0.005	0.003	−0.055	−0.011, 0.000	−1.853	0.064	1.009	0.09
	Erotophilia	0.009	0.008	0.037	−0.00, 0.024	1.113	0.266	1.271	
	Positive attitudes toward sexual fantasies	−0.049	0.006	−0.284	−0.06, −0.038	−8.586	<0.001	1.249	
	Negative attitude toward masturbation	0.034	0.016	0.067	0.003, 0.065	2.155	0.031	1.092	

Note. Higher scores for desire, arousal, vaginal lubrication, ability to have an orgasm and orgasm satisfaction, worse sexual function. B: non standardized beta; SE: standard error; β: standardized beta; 95%CI: 95% confidence interval.

**Table 4:** Hierarchical regression for sexual function in men.

Predictors		B	SE	β	95%CI	t	*p*	VIF	$R^{2}$
Model 1. Sexual desire	Age	0.003	0.002	0.037	−0.002, 0.007	1.182	0.237	1.008	0.09
	Erotophilia	−0.010	0.006	−0.058	−0.023, 0.002	−1.621	0.105	1.364	
	Positive attitudes toward sexual fantasies	−0.037	0.005	−0.245	−0.047, −0.026	−6.956	<0.001	1.302	
	Negative attitudes toward masturbation	0.020	0.010	0.065	0.000, 0.040	1.954	0.051	1.180	
Model 2. Sexual arousal	Age	0.004	0.002	0.056	0.000, 0.008	1.798	0.072	1.008	0.10
	Erotophilia	−0.010	0.006	−0.061	−0.022, −0.002	−1.693	0.091	1.364	
	Positive attitudes toward sexual fantasies	−0.038	0.005	−0.266	−0.048, −0.028	−7.585	<0.001	1.302	
	Negative attitudes toward masturbation	0.010	0.010	0.034	−0.009, 0.029	1.012	0.312	1.180	
Model 3. Erection	Age	0.022	0.002	0.298	0.018, 0.027	9.825	<0.001	1.008	0.13
	Erotophilia	−0.015	0.007	−0.081	−0.028, −0.002	−2.308	0.021	1.364	
	Positive attitudes toward sexual fantasies	−0.015	0.005	−0.097	−0.026, −0.005	−2.803	0.005	1.302	
	Negative attitudes toward masturbation	0.030	0.011	0.093	0.009, 0.051	2.819	0.005	1.180	
Model 4. Ability to have an orgasm	Age	0.001	0.002	0.014	−0.003, 0.005	0.450	0.653	1.008	
	Erotophilia	−0.023	0.006	−0.133	−0.036, −0.011	−3.655	<0.001	1.364	
	Positive attitudes toward sexual fantasies	−0.022	0.005	−0.144	−0.032, −0.011	−4.029	<0.001	1.302	
	Negative attitudes toward masturbation	0.019	0.010	0.060	−0.002, 0.039	1.773	0.077	1.180	
Model 5. Orgasm satisfaction	Age	−0.002	0.002	−0.026	−0.006, 0.003	−0.829	0.407	1.088	0.07
	Erotophilia	−0.010	0.006	−0.058	−0.022, 0.002	−1.591	0.112	1.364	
	Positive attitudes toward sexual fantasies	−0.028	0.005	−0.184	−0.038, −0.017	−5. 184	<0.001	1.302	
	Negative attitudes toward masturbation	0.036	0.010	0.117	0.016, 0.056	3.468	0.001	1.180	

Note. Higher scores for desire, arousal, erection, ability to have an orgasm and orgasm satisfaction, worse sexual function. B: non standardized beta; SE: standard error; β: standardized beta; 95%CI: 95% confidence interval.

## Data Availability

The data presented in this study are available on request from the corresponding author. The data are not publicly available due to privacy.
